# How to Soften Hard Putty

**Published:** 1873-01

**Authors:** 


### HOW TO SOFTEN HARD PUTTY.

It is well known that the common putty, with which glass window-panes are fixed in their frames, is made of powdered chalk and linseed-oil. When old, it becomes so hard that, in case its removal is necessary, a chisel and hammer must be resorted to. In fact, it becomes like a stone, harder than the wood itself, pieces of which often break off unless peculiar care is taken in removing the putty. This hardness becomes a serious inconvenience when a large pane, say of valuable plate-glass, has to be removed, for the purpose of repairs in the wood-work, or from some other cause. Here the use of chisel and hammer on the putty surrounding the glass may cause serious damage along the edges, or even fatal fracture.—An agent to soften the putty in such cases, so that it may be removed with ease, is, therefore, of some value. This may be effected with a paste of caustic potassa, easily prepared by mixing the caustic alkali, or even carbonate of potash or soda, with equal parts of freshly-burnt lime, which has previously been sprinkled with water, so as to cause it to fall into powder. This mixture is then mixed with water to a paste, and this is spread on the putty to be softened. Where one application is not sufficient, it is repeated. In order to prevent the paste from drying too quickly, it is well to mix it with less water, adding some soft-soap instead.—*Manufacturer and Builder.*

---

KEEPING FRUIT IN OUR ROOMS.—We should be chary of keeping ripe fruit in our sitting-rooms, and especially beware of laying it about a sick chamber for any length of time. The complaint which some people make about a faint sensation in the presence of fruit, is not fanciful—they may be really affected by it; for two continental chemists have shown that from the moment of plucking apples, cherries, currants, and other fruits are subject to incessant transformation. At first, they absorb oxygen, thus robbing the surrounding air ot its vital element. Then they evolve carbonic acid, and this is far greater volume than the purer gas is absorbed, so that we have poison given us in the place of pure air, with compound interest. Temperature affects the rate of changes, warmth accelerating it.

---

A POWERFUL disinfectant, especially adapted to the destruction of insects, is prepared by passing sulphuric acid into alcohol.

